# Supplementary material for: Using Drosophila melanogaster to Analyse the Human Paralogs of the ESCRT-III Core Component Shrub/CHMP4/Snf7 and Its Interactions with Members of the LGD/CC2D1 Family
Source: Int J Mol Sci. 2022 Jul 6;23(14):7507. doi: 10.3390/ijms23147507 (PMC9320689; doi:10.3390/ijms23147507)
Supplement: Supplementary file 1 [file ijms-23-07507-s001.zip › ijms-1772245-supplementary.pdf]

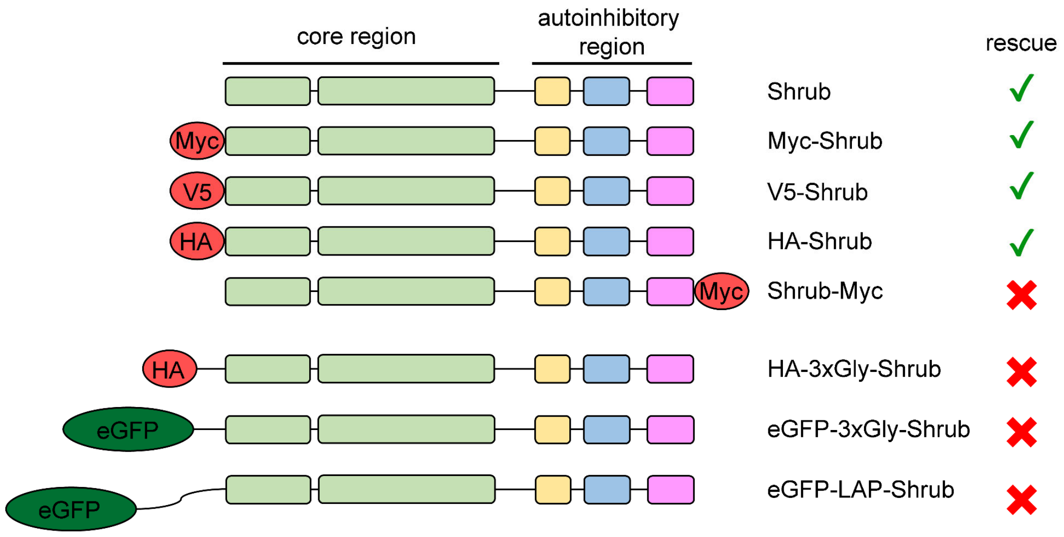

Figure S1. Tagging and its effect on Shrub functionality. Shrub consists of a core domain and an autoinhibitory C-terminal region. Highlighted in green the two crystalized helices that are essential for Shrub polymerisation [9], in yellow a domain responsible for autoinhibition, in blue the MIM domain mediating the interaction with Vps4 and in purple a helix responsible for the interaction with Bro1/Alix. On the right side the ability of the tagged Shrub variants to rescue *shrub*$^{4-1}$ (null) lethality is visualized. Red cross: no rescue; green tick: rescue.

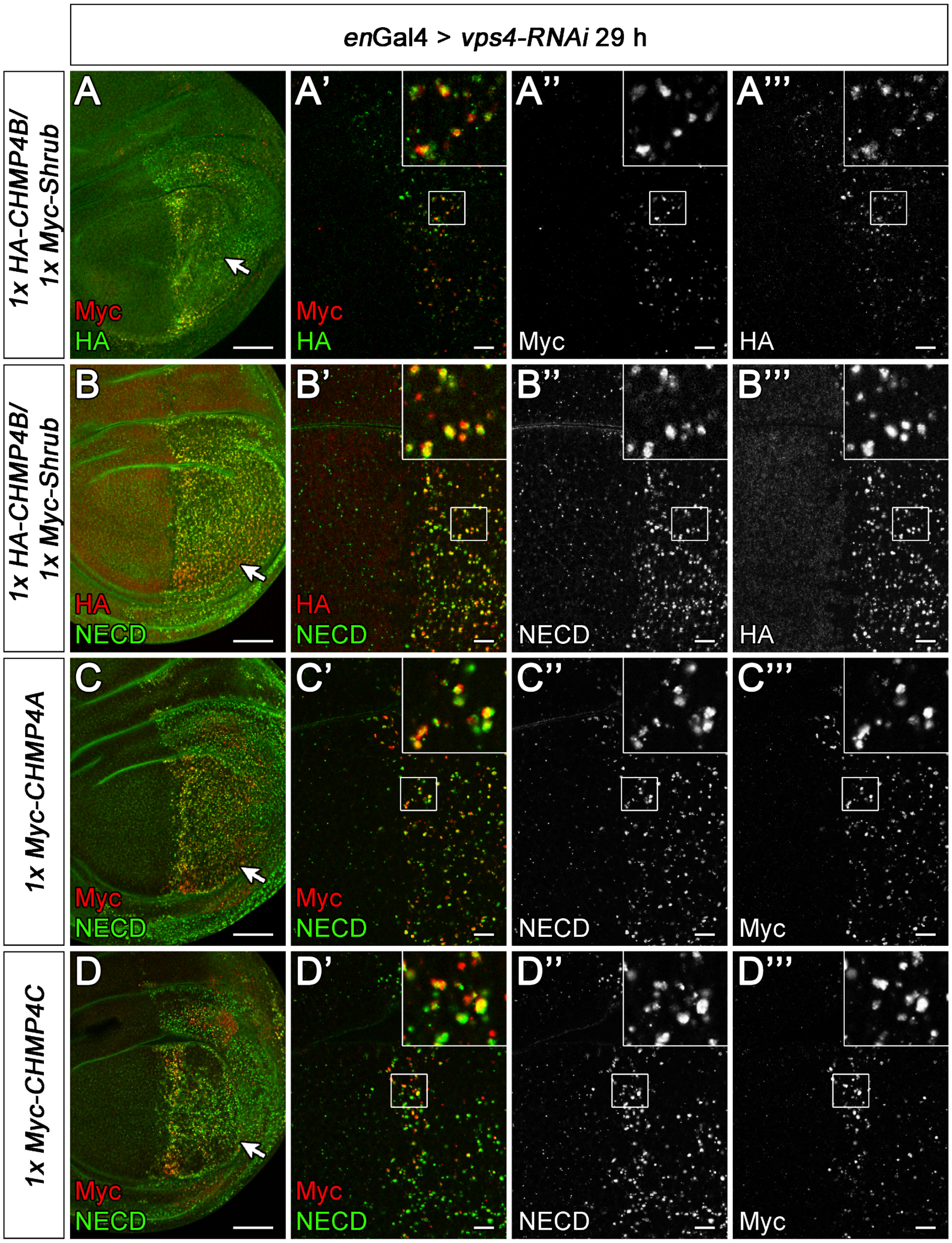

Figure S2. Human CHMP4s cycle between cytosol and the limiting membrane of endosomes. (A-B′′′) Depletion of Vps4 (RNAi) in the posterior compartment (arrow) of a wing disc using *enGal4* results in an accumulation of Myc-Shrub at enlarged Notch positive endosomes. (C-D′′′) A similar behaviour was found for Myc-CHMP4A (C-C′′′) and Myc-CHMP4C (D-D′′′). Thus, CHMP4A, CHMP4C and CHMP4B are recruited to endosomes. Scale bar (A-D) 50 μm, (A′ – D′′′) 10 μm. At least ten wing imaginal discs were analysed for each genotype.

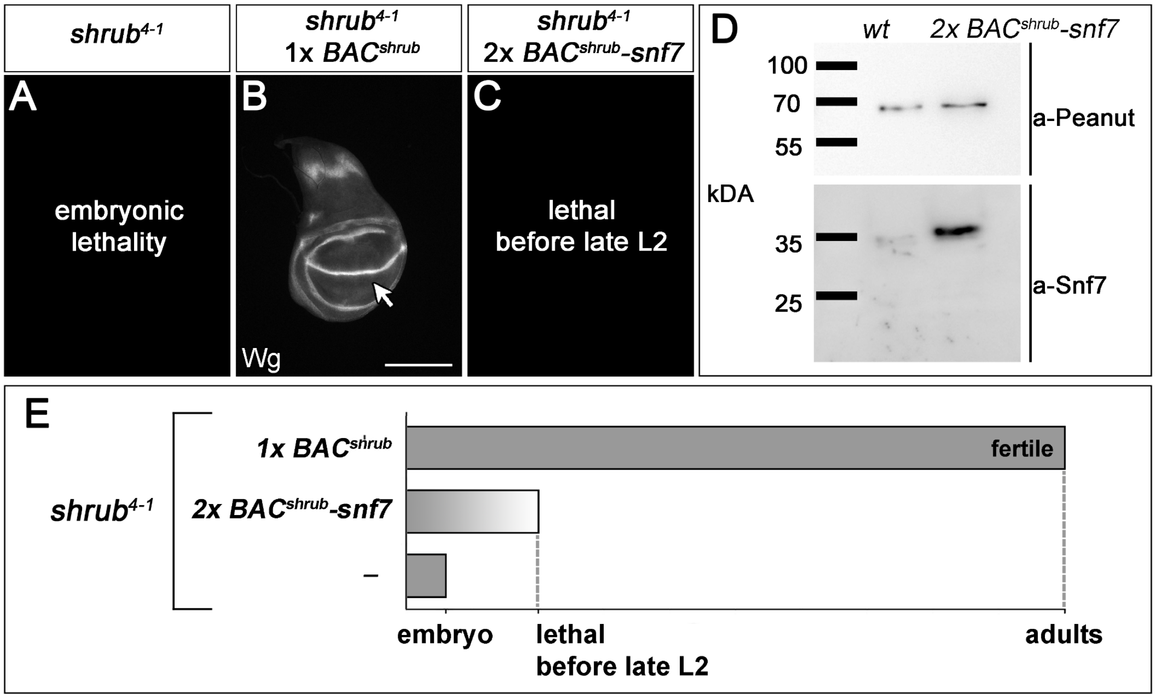

Figure S3. Snf7 is not able to rescue *shrub* mutants. (A-C) Snf7, expressed under control of *BAC^shrub^*, is not able to rescue the *shrub^4-1^* lethality. As *shrub* mutants die before the late second instar stage, even in the presence of *BAC^shrub^-snf7*. (D) A Western Blot analysis confirms expression of Snf7 (~35 kDA) in *Drosophila*. As a reference the ubiquitously expressed Peanut has been used. (E) Summary of the time of death from *shrub* rescue experiments based on the *shrub^4-1^* lethality expressing *BAC^shrub^* and *BAC^shrub^-snf7*. Scale Bar (B) 200 µm. At least ten wing imaginal discs were analysed for each genotype.

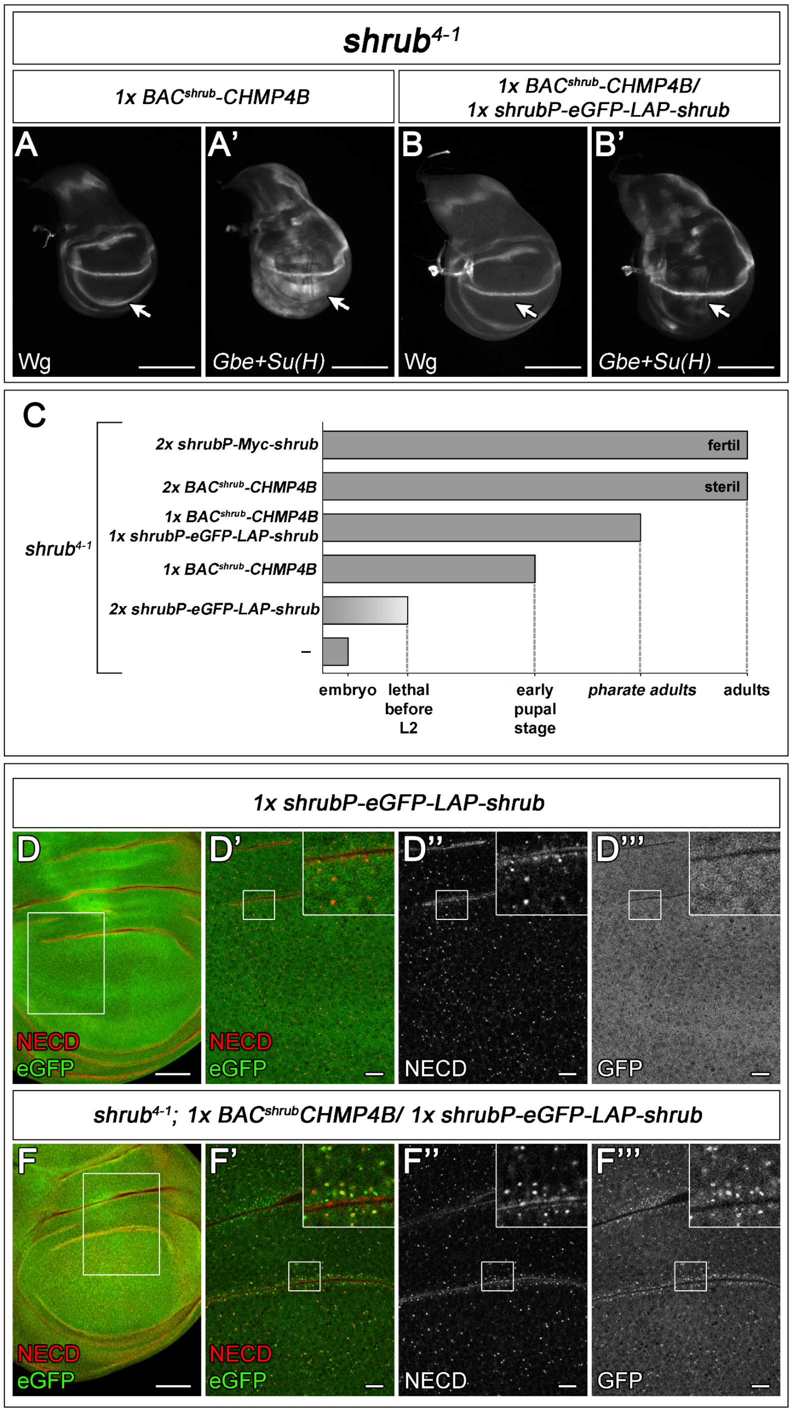

Figure S4. Determining the functionality of eGFP-LAP-shrub. (A-B') Enhancement of the rescue abilities of one copy of *BAC*^{shrub}*-CHMP4B* by *shrubP-eGFP-LAP-shrub*. (A, A') In the *shrub* mutant discs partially rescued by one copy of CHMP4B, a weak ectopic expression of Wg (arrow in A) and Gbe+Su(H) is detectable, indicating the weak ectopic activation of the Notch pathway. (B, B') The weak ectopic expression of the targets and therefore the activity of the pathway is suppressed by the addition of one copy of *shrubP-eGFP-LAP-shrub*, indicating that eGFP-LAP-Shrub can synergise with CHMP4B although it is not active if present alone. (C) Summary of the rescue experiments. eGFP-LAP-Shrub is not able to rescue shrub mutants on its own, but can enhance the rescue by CHMP4B, indicated by the shift of the time of death from the early to the late pupal stage. (D-E''') Analysis of the subcellular localization of eGFP-LAP-Shrub. (D-D''') eGFP-LAP-shrub is evenly distributed in the cytosol. (E-E''') In the presence of CHMP4B, eGFP-LAP-Shrub accumulates at the

membrane of Notch-positive endosomes. Scale bar (A-B') 200 µm, (D-F) 50 µm, (D'-F''') 10 µm. At least ten wing imaginal discs were analysed for each genotype.

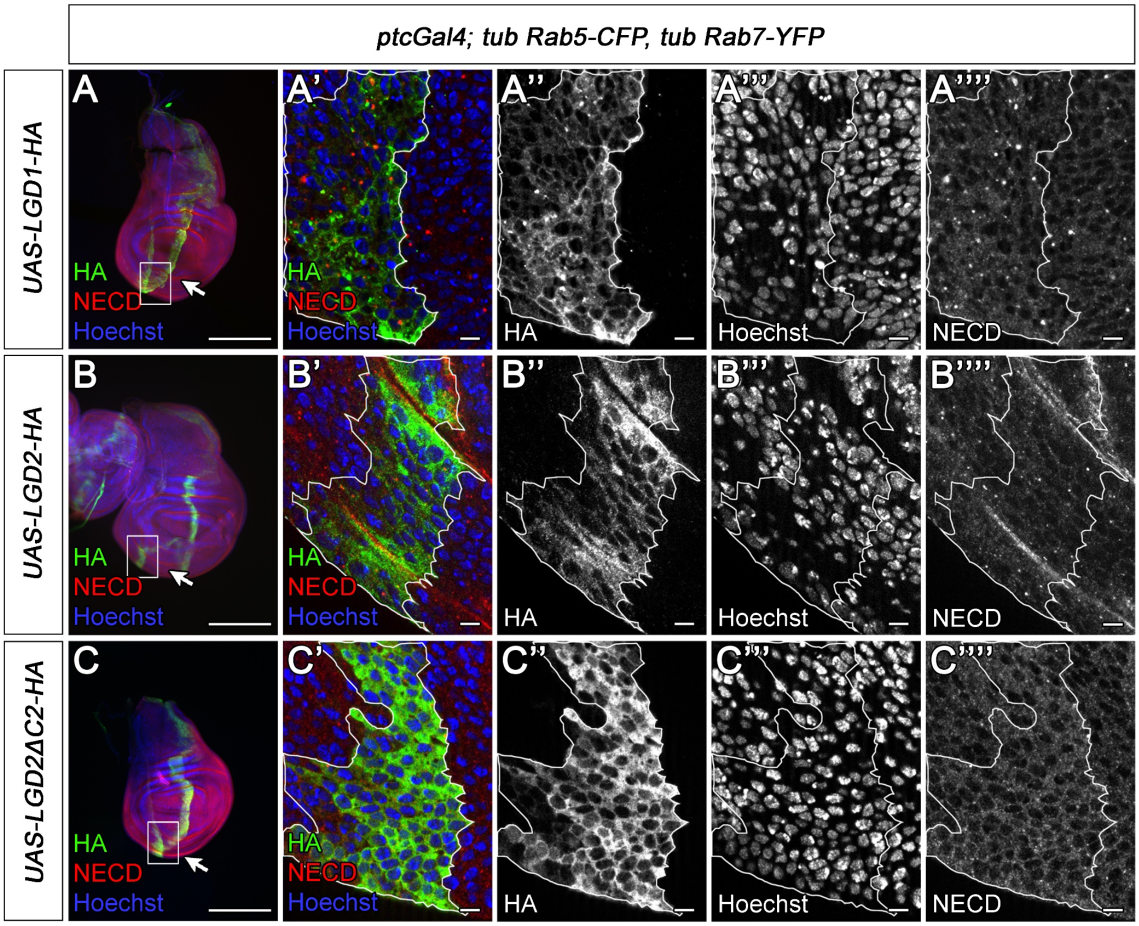

Figure S5. Human LGD1 and LGD2 are located in the cytosol, even if their C2-domain is deleted. (A-C'''') Expression of LGD1 and LGD2 with *ptcGal4*. Each imaginal disc was stained for Hoechst to visualize nuclei, for HA to detect the subcellular localisation of the indicated LGD variants and for the extracellular domain of Notch (NECD) to reveal endosomes. Scale Bar (A-D) 200 µm, (A'-D') 10 µm. At least ten wing imaginal discs were analysed for each genotype.

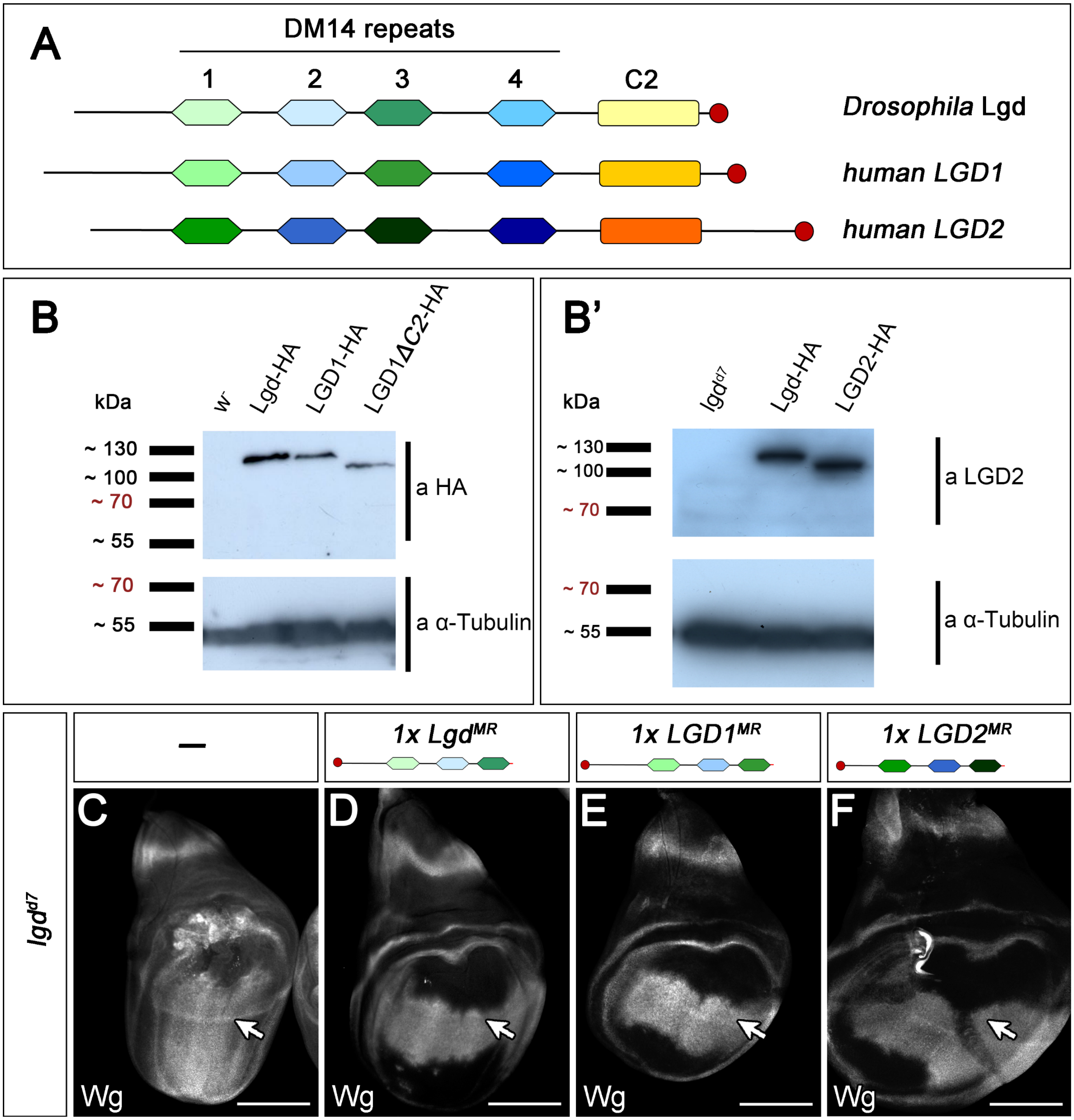

Figure S6. Validation of human LGD expression and functional analysis of LGD^{MR} variants in *Drosophila*. (A) Lgd, LGD1 and LGD2 domain architecture, highlighted the C-terminal C2-Domain and the four DM14 Domains. All LGD constructs in this study are HA-tagged at the C-Terminus (red circle). Note the longer C-terminal tail of LGD2 after its C2 domain. (B-B') Western Blot analyses confirmed a similar protein expression level of *lgdP*-lgd-HA, *lgdP*-LGD1-HA and *lgdP*-LGD2-HA. Lgd/LGD variants were detected by either HA (B) or LGD2 (B') antibody. As a reference Tubulin was used. (C-F) Rescue of *lgd^{d7}* (null) lethality by the Lgd^{MR}, LGD1^{MR} or LGD2^{MR} variants. Each imaginal disc was stained for the Notch target gene, *wingless* (*wg*) to reveal the activity of the Notch pathway. All three variants were not able to rescue the *lgd* mutant phenotype. The characteristic ectopic activation of Notch in *lgd* mutants, revealed by anti Wg staining was detected in the presence of each of the variants (D-F) and comparable to *lgd* mutants (C). Scale bar (D-F) 200 μm. At least ten wing imaginal discs were analysed for each genotype. .
